# Breathing‐driven modulation of reticulospinal tract activity

**DOI:** 10.1113/EP093536

**Published:** 2026-02-15

**Authors:** Ruqayya Thawer, Stuart N. Baker, Boubker Zaaimi

**Affiliations:** ^1^ College of Life and Health Sciences Aston University Birmingham UK; ^2^ Faculty of Medical Sciences Newcastle University Newcastle upon Tyne UK

**Keywords:** neurorehabilitation, reaction times, respiratory rhythms, reticulospinal tract, StartReact paradigm

## Abstract

Breathing rhythms influence brain activity, but whether they modulate the excitability of the reticulospinal tract (RST; a key pathway for motor control and recovery after stroke) remains unknown. In this study, we used the StartReact paradigm to examine how respiratory rhythms modulate RST excitability during motor tasks, measuring reaction times across visual, visual–auditory and visual–auditory startling conditions in three arm muscles (first dorsal interosseous, flexor digitorum superficialis and biceps) of healthy adults (*n* = 13). Reaction times decreased significantly from visual to visual–auditory to visual–auditory startling conditions. Crucially, respiratory‐phase transitions, particularly from inspiration to expiration, significantly enhanced RST excitability specifically during startle‐evoked responses, with StartReact effects being significantly stronger during respiratory transitions compared with mid‐phases (*P* ≤ 0.011). These findings suggest that respiratory rhythms modulate RST excitability dynamically in a phase‐ and condition‐specific manner. The identification of respiratory transition phases as optimal periods for RST activation could inform new neurorehabilitation strategies, such as respiratory‐phase‐aligned stimulation, to enhance motor recovery following corticospinal lesions.

## Abstract (highlights)

- **What is the central question of this study?**
  We investigated whether respiratory rhythms modulate reticulospinal tract excitability during voluntary movement.
- **What is the main finding and its importance?**
  Using the StartReact paradigm, we found that reaction times were shortest during respiratory‐phase transitions, especially from inspiration to expiration in response to startling auditory stimuli. These findings suggest that breathing rhythms influence reticulospinal drive dynamically and could inform respiratory‐phase‐aligned interventions to enhance motor recovery after stroke.

## 1 INTRODUCTION

Stroke is a leading cause of long‐term disability, often resulting in severe motor impairments (Katan & Luft, 2018). Although therapies aimed at motor recovery have traditionally focused on rehabilitative training (Dee et al., 2020), neuromodulation techniques, such as vagus nerve stimulation (VNS), are gaining attention for enhancing motor recovery (Huang et al., 2024; Keser et al., 2023; Ting et al., 2021). The mechanisms underlying VNS‐induced motor recovery remain unclear (Andalib et al., 2023; Bowles et al., 2022; Keser et al., 2023; Schambra & Hays, 2025). How can a nerve primarily involved in regulating autonomic functions, such as breathing and the transition between inspiration and expiration, contribute to stroke‐related motor recovery? One possibility is that subcortical motor circuits, particularly the reticulospinal tract (RST), might mediate VNS‐driven improvements through their role in motor control and plasticity.

The reticular formation and its descending motor pathway, the RST, play a crucial role in motor control (Davidson & Buford, 2006; Ortiz‐Rosario et al., 2014; Riddle et al., 2009). Our prior research has demonstrated that the RST markedly increases its contribution to spinal motoneurons, compensating for lost corticospinal input, after damage to the corticospinal tract (Baker & Perez, 2017; Zaaimi et al., 2012, 2018b). However, this RST input is imbalanced, disproportionately favouring flexor over extensor motor neurons. Such imbalanced plasticity contributes to characteristic post‐stroke motor impairments, including extensor weakness and flexor spasticity. Additionally, we have shown that the reticular formation undergoes significant reorganization after corticospinal tract injury, with altered neuronal firing (Zaaimi et al., 2018b). Recent studies have highlighted the potential of targeting the RST to improve motor function after stroke (Choudhury et al., 2020). However, the factors that influence RST activity and how they can be harnessed for therapeutic purposes remain poorly understood.

Respiration, a fundamental physiological process, is increasingly recognized as a modulator of brain function. It has been demonstrated that breathing drives oscillations of the membrane potential of neurons in various cortical areas, including prefrontal and somatosensory cortices (Juventin et al., 2023). Li and Rymer (2011) examined the relationship between respiration and motor activity by using transcranial magnetic stimulation (TMS) over the motor cortex or electrical stimulation of hand muscles and discovered a general enhancement of the motor system related to breathing. The authors observed that delivering stimulation to finger extensors during voluntary inspiration resulted in a significant decrease in finger flexor spasticity in a stroke patient.

The transition from inspiration to expiration and vice versa is crucial because these shifts in the physiological state of the body can modulate cortical excitability. Zelano et al. (2016) demonstrated that the act of inhaling through the nose significantly increases delta oscillations and enhances cognitive functions, such as memory recall, directly linking the respiratory phase to cortical activity. Conversely, Nakamura et al. (2018) suggested that the transition from expiration to inspiration might affect reaction time and recall accuracy in a delayed match‐to‐sample visual recognition task. Their results showed that when this transition occurred between image presentation and response, subjects experienced significantly longer response times and decreased recall accuracy in comparison to trials where this transition did not occur.

To probe the relationship between respiration and RST function, we turn to the StartReact paradigm. This involves a shortened reaction time to an unexpected loud auditory stimulus (Valls‐Solé et al., 1999, 1995) and provides a valuable tool for investigating subcortical motor pathways (Baker & Perez, 2017; Carlsen et al., 2004, 2011, 2012). It is a well‐established behavioural method that serves as a robust, albeit indirect, proxy for assessing the excitability of subcortical motor pathways, including the RST. Given that respiratory‐phase transitions modulate cortical and brainstem excitability (Nakamura et al., 2018; Tapia et al., 2022; Zelano et al., 2016) and that StartReact responses are mediated, in part, through reticulospinal pathways, we reasoned that respiratory phase might influence StartReact measures of RST excitability specifically. If the therapeutic effect of VNS stems from leveraging respiration‐driven modulation of RST excitability, we hypothesize that the transition from inspiration to expiration (a crucial phase regulated by the inspiratory off‐switch function of the vagus nerve; von Euler & Trippenbach, 1975) could represent a period of heightened RST output. Consequently, we expect shorter reaction times for startling auditory stimuli occurring during these respiratory transition phases. This would provide indirect, yet compelling evidence for respiratory‐phase‐specific modulation of reticulospinal excitability.

## 2 MATERIALS AND METHODS

### 2.1 Ethical approval

This study was approved by the Human Research Ethics Committee at Aston University (approval no. HLS21072). All participants provided written informed consent prior to participation. The study conformed to the standards set by the latest revision of the *Declaration of Helsinki*, except for registration in a database.

### 2.2 Participants

Thirteen healthy participants (age range, 20–22; 3 males and 10 females) were recruited for this study. Eligibility was determined through a prescreening questionnaire to exclude individuals with any history of neurological disorders.

### 2.3 Electromyography

Participants were seated comfortably in a soundproof room designed to meet clinical auditory standards. Adhesive surface electrodes (model H59P Kendall; Covidien, Dublin, Ireland) were positioned over the flexor digitorum superficialis (FDS), first dorsal interosseous (1DI) and biceps muscles. For the FDS and biceps, the electrodes were spaced 2–3 cm apart, and for the 1DI the spacing was 1 cm. Signals were amplified using a D360 amplifier (Digitimer Ltd, Welwyn Garden City, UK) with a gain of 1000 and a bandpass filter ranging from 30 Hz to 2 kHz. The amplified signals were digitized via a Micro1401 interface at a sampling rate of 5 kHz and recorded using Spike2 software (Cambridge Electronic Design, Cambridge, UK).

### 2.4 Breathing measurements

Breathing signals were measured using a surgical mask equipped with a temperature sensor (LM34, Texas Instruments) positioned below the nostrils. The mask was adjusted to avoid direct skin contact with the sensor while ensuring comfort for the participants. The signals from the sensor were amplified with a gain of 500 and bandpass filtered between 0.3 and 3.4 Hz using a custom‐built circuit. The filtered signals were then digitized via the Micro1401 interface for further analysis.

### 2.5 StartReact protocol

The StartReact protocol was adapted from the methods described by Baker and Perez (2017). A red LED positioned 1 m away from the participants served as the visual cue. Participants rested their right forearm on an armrest at an angle of 90° and were instructed to perform a power grip and to flex their elbow and wrist as quickly as possible in response to the LED flash, which lasted for 20 ms. This multi‐joint task, involving elbow flexion, wrist flexion and power grip, was selected to assess coordinated motor output across proximal and distal muscles, reflecting functional upper‐limb movement patterns relevant to daily activities and rehabilitation contexts. Three conditions were presented in randomized order: a visual reaction time (VRT) condition using the LED light only; a visual–auditory reaction time (VART) condition combining the LED light with a quiet sound (80 dB, 500 Hz for 20 ms); and a visual–startle reaction time (VSRT) condition combining the LED light with a startling sound (115 dB, 500 Hz for 20 ms). Reaction times were measured as the interval between the LED cue and the onset of EMG activity. Auditory stimuli were amplified (Yamaha A‐S201) and delivered via speakers (Fenton SHFB65 HiFi Bookshelf Speakers, 200 W) to ensure consistent sound pressure levels.

Participants were familiarized with the task before the main experiment and instructed on all conditions to ensure proper understanding. To acclimate them to the startling stimulus, five loud sounds were presented at 5 s intervals without requiring any task performance. The experiment consisted of four blocks, with each block containing 20 trials for each condition (VRT, VART and VSRT) in random order, resulting in 240 trials in total (20 trials × 3 conditions × 4 blocks). The intervals between trials varied randomly between 12.5 and 15.7 s to minimize predictability and anticipation. Participants were provided with a 5 min break between blocks to minimize fatigue.

### 2.6 EMG processing and reaction time calculation

Reaction time was defined as the interval between the visual cue onset and the first detectable increase in EMG activity above baseline. This was determined using a semi‐automated approach. Initial detection used a threshold‐based algorithm (threshold set 3SD above baseline mean activity), followed by visual inspection to verify each onset. EMG signals were rectified before analysis.

### 2.7 Respiratory‐phase categorization

**FIGURE 1:**
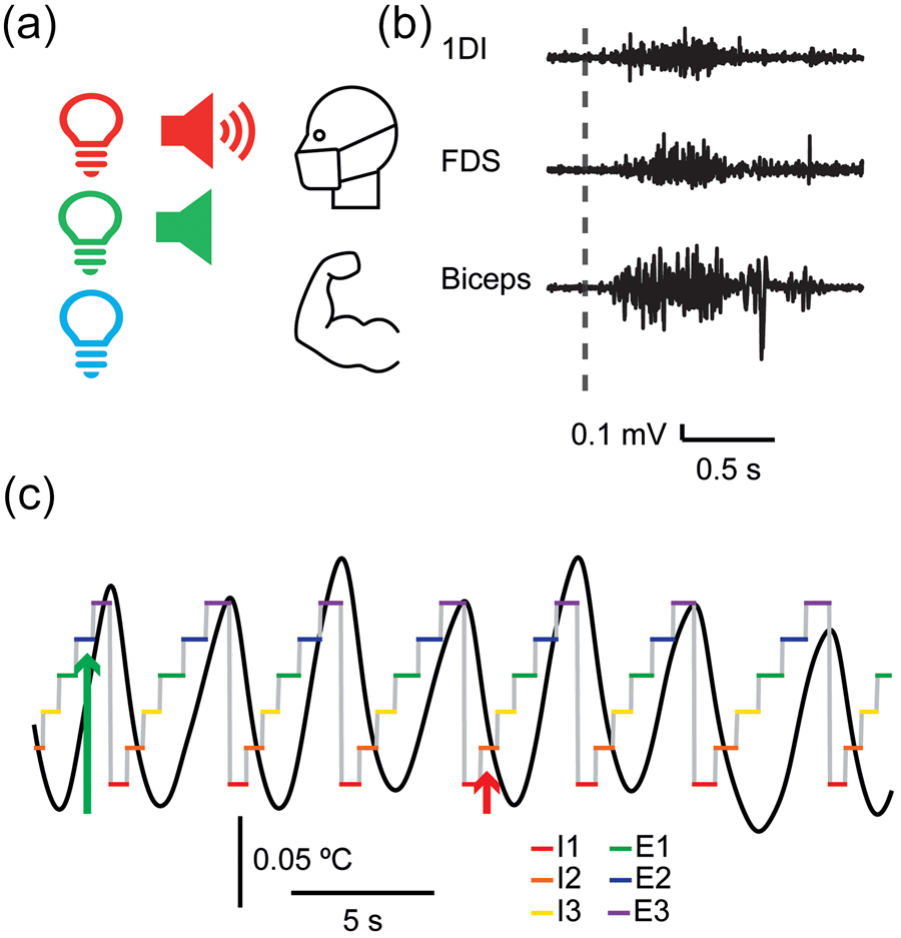
Recording of the StartReact experiment. (a) The participant was equipped with a mask containing a sensor to monitor respiratory signals and was instructed to perform an elbow flexion and a power grip as soon as the LED light was activated. The light could be delivered alone (blue condition; VRT) or accompanied by a quiet sound (green condition; VART) or by a loud, startling sound (red condition; VSRT). (b) All conditions were presented in a randomized order while recording the respiratory signal, in addition to EMG activity from the first dorsal interosseous (1DI), flexor digitorum superficialis (FDS) and biceps muscles. (c) The respiratory signal (black trace) was segmented into six distinct phases, with a decrease in temperature during inspiration and an increase during expiration. Both the inspiration and expiration cycles were divided into three equidistant phases: start of inspiration (red, I1), middle of inspiration (orange, I2), end of inspiration (yellow, I3), start of expiration (green, E1), middle of expiration (blue, E2) and end of expiration (magenta, E3). Stimulations were categorized according to the respiratory phase in which they occurred. In this example, a VART stimulation (green arrow) occurs during the middle of expiration (E2) and a VSRT stimulation (red arrow) occurs during the middle of inspiration (I2).

The respiratory signals recorded during the experiment were segmented into inspiration and expiration periods. Each period was further divided into three equidistant phases, resulting in a total of six phases: the start (I1), middle (I2) and end of inspiration (I3), in addition to the start (E1), middle (E2) and end of expiration (E3). Stimuli delivered during the experiment were categorized based on the respiratory phase at the time of occurrence to examine phase‐specific effects on reaction times. Figure 1 illustrates the experimental set‐up and the process of respiratory signal analysis.

### 2.8 Analysis

Reaction times for the three conditions (VRT, VART and VSRT) were assessed for normality using the Kolmogorov–Smirnov test in MATLAB. The test indicated significant deviations from normality (*P* < 0.05), hence non‐parametric tests were used instead of parametric ANOVA. The Friedman test, used for within‐subject phase comparisons, is the non‐parametric equivalent of repeated‐measures ANOVA. Differences across conditions were analysed using the Mann–Whitney *U*‐test, and VART–VSRT differences from zero were assessed using the one‐sample Wilcoxon signed‐rank test.

To assess whether peaks and troughs in reaction times across respiratory phases were statistically significant, a model‐based approach was used. We fitted a model made up of a mixture of two sinusoids to the reaction time data (*T*) as a function of the phase of respiration *x* (varying from 1 to 6; 1 indicates the onset of inspiration and 4 the onset of expiration):

$$T\left(x\right)=k+A_{1}\sin\left(\frac{2\pi \left(x-1\right)}{6}+\theta_{1}\right)+A_{2}\sin\left(2\frac{2\pi \left(x-1\right)}{6}+\theta_{2}\right)$$

Here, *k* is a constant offset, *A*_1_ and *A*_2_ are the sinusoid amplitudes, and *θ*_1_ and *θ*_2_ are phase shifts (in radians).

For primary analysis and sinusoidal fitting, reaction times were grouped by respiratory phase using all available trials. To characterize data variability, SD was used throughout. Individual data points are displayed in all figures to show the distribution of participant‐level means. The StartReact effect was calculated as the difference between the VART and VSRT for each respiratory phase and each muscle. To evaluate the significance of observed peaks and troughs in individual reaction time measures, or the StartReact effect, simulations were performed. A bootstrapping approach was used to generate 10 000 simulated datasets by randomly shuffling reaction times across respiratory phases. For each simulated dataset, sinusoidal fits were computed. Peaks at the transition phases between inspiration and expiration (I3–E1) and troughs at the middle of inspiration (I2) and the middle of expiration (E2) were extracted from the sinusoidal fits to both the real and simulated data. These phases were selected based on previous literature suggesting that motor excitability is particularly sensitive to changes during the transition from inspiration to expiration, when respiratory‐related modulation is most pronounced. Conversely, a relative suppression of effects could be expected during the middle of inspiration or expiration, potentially reflecting more stable respiratory‐driven motor states. The measured peak or trough was compared with the simulated distributions, and the *P*‐value was calculated as the proportion of simulations that exceeded (for peaks) or fell below (for troughs) the measured value.

Respiratory‐phase modulation was assessed using multiple approaches: (1) sinusoidal fits modelling reaction time modulation across six respiratory phases; (2) Friedman tests (non‐parametric repeated‐measures ANOVA equivalent) to assess main effects of respiratory phase; and (3) comparison of phase modulation strength between conditions using Wilcoxon signed‐rank tests on the SD across phases.

StartReact effect analysis (VART–VSRT difference) included: (1) bootstrapping with 10 000 simulations to assess significance of phase‐specific peaks and troughs; (2) focused comparisons between respiratory transition phases (I3–E1) and mid‐phases (I2, E2) using Wilcoxon rank‐sum tests; and (3) specific testing of inspiration–expiration transition enhancement. Exact *P*‐values are reported for all tests. Statistical significance was set at *P* < 0.05.

## 3 RESULTS

**FIGURE 2:**
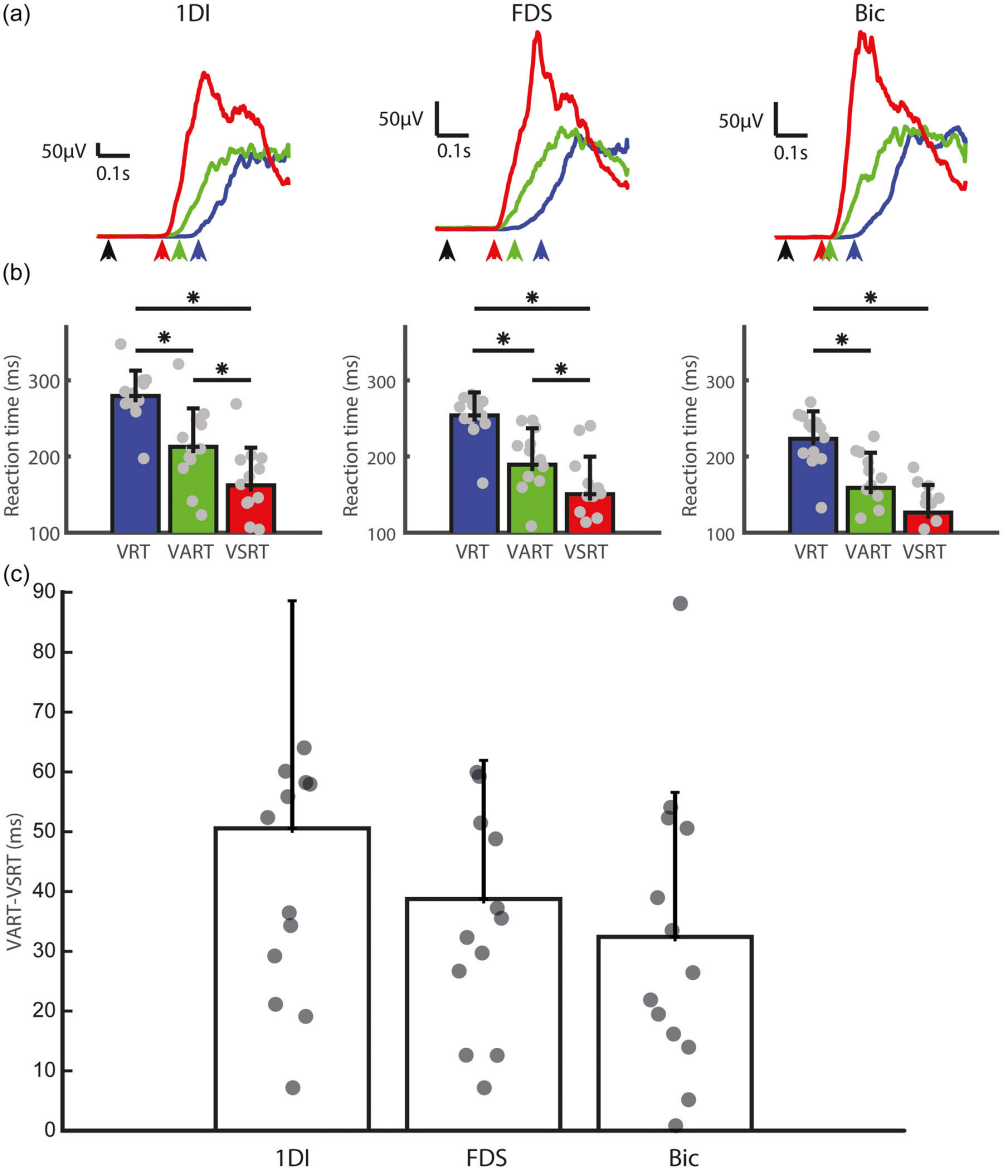
Rectified EMG traces, reaction times and StartReact effects for VRT, VART and VSRT conditions across muscles. (a) Rectified EMG traces for the first dorsal interosseous (1DI, left), flexor digitorum superficialis (FDS, middle) and biceps (Bic, right) muscles during the VRT (blue), VART (green) and VSRT (red) conditions in a representative participant. Reaction times shortened during the VSRT condition compared with the VART and VRT conditions across all muscles. The black arrows indicate the time of the cue, and the blue, green and red arrows show the mean reaction times for the VRT, VART and VSRT conditions, respectively. (b) Bar charts showing reaction times for the VRT, VART and VSRT conditions across the 1DI, FDS and biceps muscles. Data are presented as the mean + SD. Individual data points from all participants (*n* = 13) are overlaid. Asterisks indicate statistically significant differences based on the Mann–Whitney *U*‐test (*P < 0.05, **P < 0.01 and ***P < 0.001). (c) StartReact effect (VART–VSRT difference) across muscles. Data are presented as the mean + SD, with individual data points overlaid. One‐sample Wilcoxon signed‐rank tests confirmed that VART–VSRT differences were significantly greater than zero for all muscles (1DI, *P* = 0.0002; FDS, *P* = 0.0002; biceps, *P* = 0.0002). Rectified EMG traces, reaction times and StartReact effects for visual reaction time (VRT), visual–auditory reaction time (VART) and visual–startle reaction time (VSRT) conditions across muscles.

Figure 2a displays the average rectified EMG traces for the 1DI, FDS and biceps muscles across the VRT, VART and VSRT conditions for a single subject. In all muscles, the VSRT condition showed the shortest reaction times compared with both the VRT and VART conditions, indicating that the VSRT condition produced faster motor responses.

Figure 2b shows that for the 1DI, reaction times were significantly shorter in the VSRT condition compared with both VART (162 ± 49 vs. 213 ± 50 ms; Mann–Whitney *U*‐test, *P* = 0.0103) and VRT (162 ± 49 vs. 280 ± 33 ms; *P* = 0.0001). Likewise, VART was significantly shorter than VRT (213 ± 50 vs. 280 ± 33 ms; *P* = 0.0010). For the FDS, reaction times were shortest in the VSRT condition compared with VART (151 ± 49 vs. 189 ± 48 ms; *P* = 0.0402) and VRT (151 ± 49 vs. 254 ± 30 ms; *P* < 0.0001). VART was also shorter than VRT (189 ± 48 vs. 254 ± 30 ms; *P* = 0.0004). For the biceps, reaction times were shorter in the VSRT condition compared with VRT (126 ± 36 vs. 223 ± 36 ms; *P* = 0.0001). Although there was a reduction in RT from VART to VSRT, this difference did not reach statistical significance (126 ± 36 vs. 159 ± 46 ms; *P* = 0.0513). VART was significantly shorter than VRT (159 ± 46 vs. 223 ± 36 ms; *P* = 0.0012).

Figure 2c illustrates the VART–VSRT difference (StartReact effect) across muscles. One‐sample Wilcoxon signed‐rank tests confirmed that the VART–VSRT difference was significantly greater than zero for all three muscles: 1DI (mean = 51 ± 38 ms; *P* = 0.0002), FDS (mean = 39 ± 23 ms; *P* = 0.0002) and biceps (mean = 32 ± 24 ms; *P* = 0.0002). Pairwise comparisons between muscles using the Mann–Whitney *U*‐test revealed no significant differences: 1DI versus FDS (*P* = 0.4119), 1DI versus biceps (*P* = 0.0906) and FDS versus biceps (*P* = 0.4728).

**FIGURE 3:**
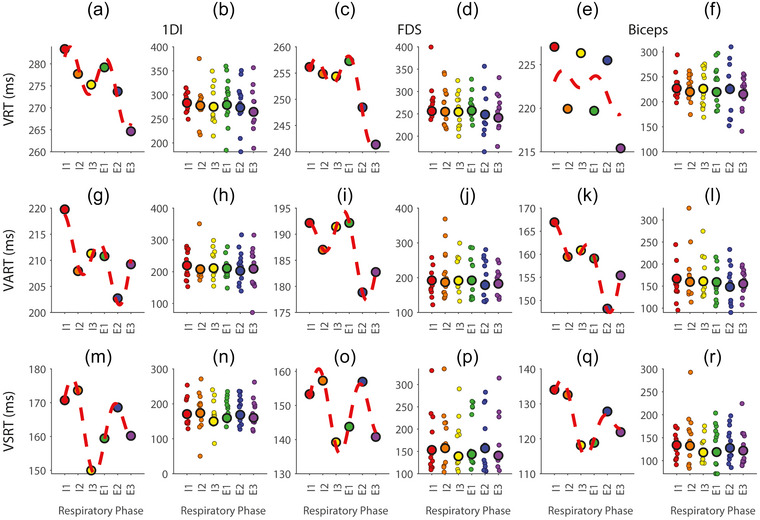
Respiratory‐phase modulation of reaction times across conditions and muscles. (a, c, e) Mean reaction times with sinusoidal fits for 1DI, FDS and biceps muscles in the VRT condition. (b, d, f) Individual participant data distributions for the VRT condition. (g, i, k) VART condition means with fits. (h, j, l) Individual participant data distributions for the VART condition. (m, o, q) The VSRT condition means with fits. (n, p, r) Individual participant data distributions for the VSRT condition. Respiratory phases: I1–I3, inspiration; and E1–E3, expiration.

Figure 3 illustrates sinusoidal fits modelling the modulation of reaction times (RTs) across six respiratory phases for the three experimental conditions. The six respiratory phases represent the start, middle and end of inspiration (I1, I2 and I3) and expiration (E1, E2 and E3), with the inspiration–expiration transition occurring between I3 and E1.

In the VRT condition (Figure 3a,c,e), the sinusoidal fit revealed minimal modulation. For the 1DI muscle, the modulation amplitude was 3.2 ms, with no significant peaks or troughs across respiratory phases (all *P* ≥ 0.127). The FDS muscle showed a modulation amplitude of 4.5 ms, with no significant phase‐dependent modulation (all *P* ≥ 0.136). Likewise, the biceps muscle displayed minimal modulation amplitude of 3.5 ms, with no significant respiratory‐phase effects (all *P* ≥ 0.121). These findings indicate weak, non‐significant respiratory‐phase‐related modulation in the VRT condition.

In the VART condition (Figure 3g,i,k), in which a quiet auditory cue was added, sinusoidal fits indicated slightly stronger but still non‐significant modulation. For the 1DI muscle, the modulation amplitude was 6.4 ms, with no significant peaks or troughs (all *P* ≥ 0.129). The FDS muscle showed minimal modulation (amplitude = −1.7 ms), with no significant phase effects (all *P* ≥ 0.424). The biceps muscle displayed a modulation amplitude of 2.2 ms, with no significant respiratory‐phase differences (all *P* ≥ 0.148).

In the VSRT condition (Figure 3m, o, q), the startling auditory stimulus elicited the strongest RT modulation. For the 1DI muscle, the sinusoidal fit showed significant modulation (amplitude = 14.4 ms), with a significant trough during the inspiration–expiration transition (I3–E1; *P* = 0.025). The FDS muscle showed significant modulation (amplitude = 9.3 ms), with a significant trough at the inspiration–expiration transition (*P* = 0.036). The biceps muscle displayed modulation (amplitude = 8.6 ms), with a trend towards significance at the inspiration–expiration transition (*P* = 0.055). These results suggest that the respiratory phase significantly modulates reticulospinal excitability during startle‐evoked responses, with the strongest effects occurring during transitions between inspiration and expiration.

Analysis of individual reaction time distributions (Figure 3, right panels) revealed substantial variability, with SDs of phase modulation ranging from 47 to 89 ms across conditions and muscles. Despite this considerable trial‐to‐trial variability, the consistent respiratory‐phase modulation patterns evident in the mean data (Figure 3, left panels) demonstrate robust phase‐dependent effects. The significant enhancement of phase modulation in VSRT compared with VRT conditions (*P* ≤ 0.027 for hand muscles) indicates that respiratory influences on reticulospinal excitability are sufficiently strong to overcome inherent motor variability.

**FIGURE 4:**
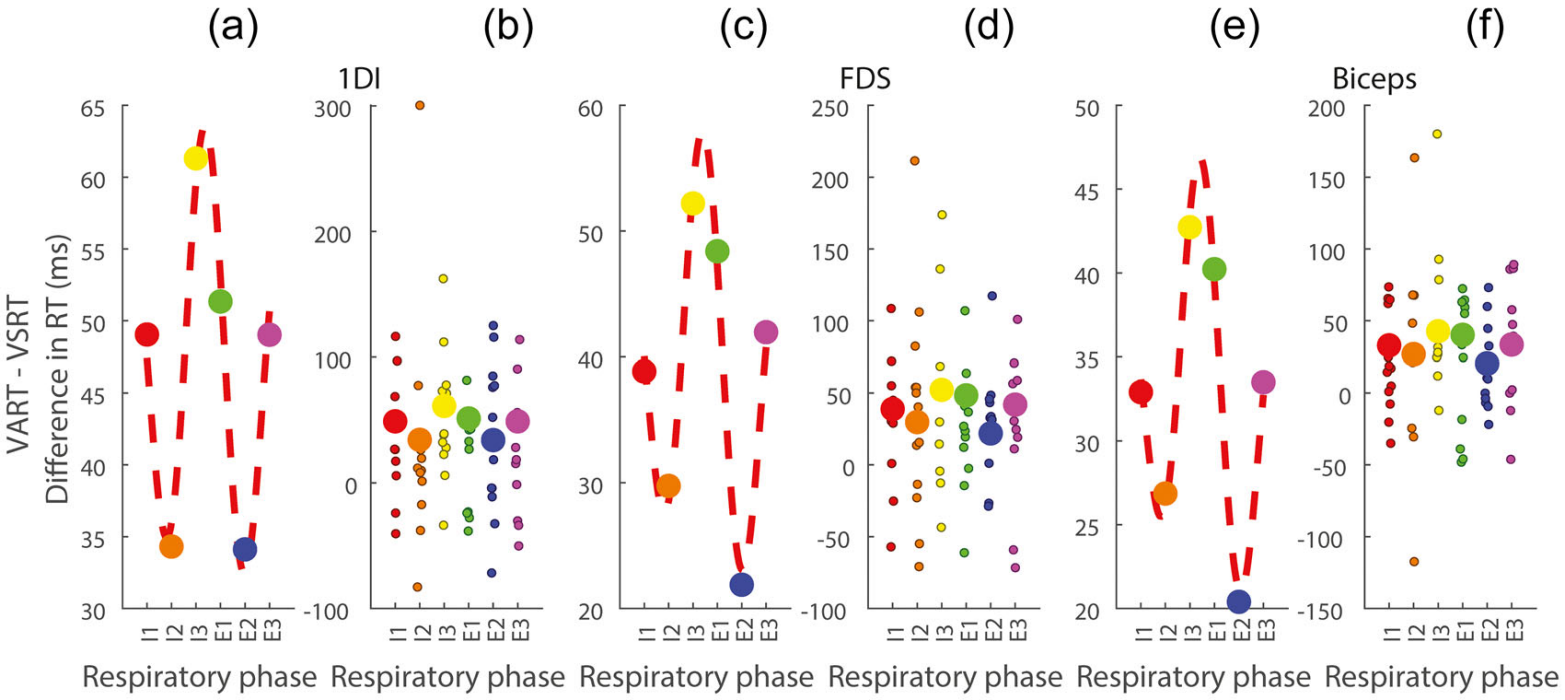
Respiratory‐phase modulation of the StartReact effect across muscles. (a, c, e) Mean StartReact effect (VART–VSRT difference) with sinusoidal fits for the first dorsal interosseous (1DI; a), flexor digitorum superficialis (FDS; c) and biceps (e) muscles. (b, d, f) Individual participant data distributions showing all VART–VSRT difference values for the corresponding muscles. Significant enhancement of the StartReact effect was observed during respiratory‐phase transitions compared with mid‐phases (1DI, *P* = 0.037; FDS, *P* = 0.003; biceps, *P* = 0.011). Respiratory phases: I1–I3, inspiration; and E1–E3, expiration.

Figure 4 illustrates the StartReact effect (VART–VSRT difference) across respiratory phases. Direct comparison of respiratory transition phases (I3–E1) versus mid‐phases (I2 and E2) revealed significantly enhanced StartReact effects during transitions for all three muscles (1DI, *P* = 0.037; FDS, *P* = 0.003; biceps, *P* = 0.011). The inspiration–expiration transition specifically showed significant enhancement for the FDS (*P* = 0.014), with trends observed for the other muscles (1DI, *P* = 0.205; biceps, *P* = 0.089).

Supporting these findings, bootstrapping analysis demonstrated significant phase‐specific modulation, with peaks during the inspiration–expiration transition (FDS, *P* = 0.043) and troughs during mid‐expiration (1DI, *P* = 0.029; FDS, *P* = 0.011; biceps, *P* = 0.030). Although Friedman tests assessing overall differences across all six respiratory phases showed trends but did not reach significance (all *P* ≥ 0.072), the specific hypothesis‐driven comparisons provide strong evidence that respiratory transitions, particularly the inspiration–expiration transition, significantly enhance reticulospinal excitability as measured by the StartReact effect.

## 4 DISCUSSION

This study suggests that respiratory rhythms influence RST excitability dynamically, as evidenced by changes in RTs during the StartReact paradigm. We found that respiratory‐phase transitions, particularly from inspiration to expiration, significantly enhance reticulospinal drive, with the strongest effects observed during startle‐evoked responses. These findings reveal a direct link between respiratory‐phase transitions and RST facilitation. Although our sample size (*n* = 13) is consistent with within‐subject designs and revealed robust effects, these findings should be considered preliminary and warrant confirmation in larger cohorts.

Our results shown in Figure 2 align with previous studies (Choudhury et al., 2019; Dean & Baker, 2016; Honeycutt et al., 2013; Maslovat et al., 2021, 2023; Nonnekes et al., 2014; Sangari & Perez, 2019, 2020; Singh et al., 2018; Škarabot et al., 2022; Smith et al., 2019; Tapia et al., 2022; van Lith et al., 2018; Walker et al., 2024), demonstrating a reduction in RTs across the conditions. The longest RTs were observed in response to unisensory visual stimuli (VRT). These RTs decreased when a quiet sound was presented alongside the visual stimuli (VART) and were reduced further when a startling sound accompanied the visual stimuli (VSRT). In this study, participants performed a multi‐joint coordinated movement involving elbow flexion (biceps), wrist flexion (FDS) and a power grip (1DI). The RT reductions observed from VART compared with VRT, and from VSRT compared with both VART and VRT, were consistent across all three muscles.

The key new finding of this study is the respiratory‐phase‐dependent modulation of RST excitability. Although the respiratory phase showed minimal influence on visually triggered responses (VRT), it exerted powerful modulation on startle‐evoked responses (VSRT). Direct comparison revealed significantly stronger phase modulation in VSRT compared with VRT conditions across all muscles (1DI, *P* = 0.027; FDS, *P* = 0.002), with particularly enhanced StartReact effects during respiratory transitions versus mid‐phases (1DI, *P* = 0.037; FDS, *P* = 0.003; biceps, *P* = 0.011). This transition‐specific enhancement was most pronounced for the inspiration–expiration transition in the forearm flexor (FDS, *P* = 0.014), demonstrating that respiratory phase does not modulate motor pathways uniformly, but instead exerts its strongest effects during specific physiological transition points. Although the forearm flexor (FDS) showed the strongest respiratory‐phase modulation, the biceps displayed a weaker but still notable pattern. This might reflect differential reticulospinal innervation patterns or task‐specific engagement, because the power grip task probably placed greater emphasis on distal hand and forearm muscles, potentially enhancing respiratory modulation in those muscle groups. It is notable that the biceps showed the smallest StartReact effect overall in this task (Figure 2c). These muscle‐specific differences warrant further investigation in tasks that engage proximal and distal musculature more equally. Thus, the strong respiratory modulation in FDS is likely to reflect both its role in the power grip task and potentially greater respiratory‐phase sensitivity of forearm flexor motoneurons via RST pathways, whereas the biceps, although anatomically more proximal, might have been less critically engaged or differentially modulated in this specific task context.

A key consideration in interpreting our findings is the indirect nature of the RST assessment. The StartReact paradigm, although a well‐validated and widely used behavioural proxy for subcortical motor pathway excitability (Baker & Perez, 2017; Carlsen et al., 2012; Tapia et al., 2022), does not constitute a direct measure of RST activity. Our conclusion that the respiratory phase modulates RST output is therefore inferential, based on the established neurophysiological framework of the startle circuit. This is a common limitation in human studies, where direct recordings from the brainstem are not feasible. Direct evidence for RST function and its plasticity has come primarily from animal models, including our own work involving direct electrophysiological recordings from identified reticulospinal neurons in non‐human primates (Fisher et al., 2021; Zaaimi et al., 2018a, 2018b). For instance, non‐invasive brainstem recordings (e.g., functional MRI of the medulla) or electrophysiological techniques, such as TMS of the motor cortex with specific conditioning protocols such as the paired loud acoustic stimulation (LAS)–TMS protocol recently shown to induce plasticity in corticoreticular connections (Germann et al., 2023), could provide more definitive evidence in humans. Alternative approaches to probe corticoreticulospinal pathways in humans include TMS‐based methods such as those described by Ziemann et al. (1999) and Taga et al. (2021). Furthermore, animal models allowing for direct electrophysiological recordings from reticulospinal neurons during controlled respiration would be invaluable for elucidating the precise neural mechanisms underlying this respiratory–motor coupling. Finally, although our respiratory measures captured phase transitions effectively, future studies could use more granular techniques (e.g., spirometry) to explore the relationship between specific respiratory parameters and RST excitability. Additionally, although our sample size of *n* = 13 is consistent with similar within‐subject psychophysiological studies and provided sufficient statistical power for the effects reported, replication in larger cohorts would strengthen these observations.

This transition‐specific modulation aligns with the crucial role of respiratory‐phase transitions in overall physiological regulation. The RTs observed in the VART condition compared with the VRT condition align with the well‐documented redundant signals effect, wherein bisensory stimuli elicit faster responses than their unisensory components (Hershenson, 1962; Nidiffer et al., 2016; Schröger & Widmann, 1998). Traditionally, this speeding effect has been attributed to co‐activation mechanisms, whereby neural integration of unisensory inputs enhances sensorimotor activation (Miller, 1982; Molholm & Foxe, 2010). However, recent findings by Shaw et al. (2020) challenge this interpretation, suggesting that much of the redundant signals effect observed in mixed‐design protocols might result from modality switching and mixing costs. Specifically, frequent alternation between unisensory (e.g., visual) and bisensory (e.g., audiovisual) trials can slow RTs for unisensory conditions, such as VRT, thereby amplifying the perceived facilitation in bisensory conditions, such as VART (Wylie et al., 2009).

The consistent RT reductions in the startling condition VSRT compared with VRT and VART, across the biceps, FDS and 1DI muscles support the idea that RST acts as a common pathway for distributing motor commands to both proximal and distal muscles (Peterson et al., 1979; Riddle et al., 2009; Zaaimi et al., 2018a). This mechanism enables rapid, coordinated multi‐muscle responses, consistent with prior evidence of startle‐evoked reliance on subcortical circuits (Carlsen et al., 2004; Nonnekes et al., 2014; Smith et al., 2019; Valls‐Solé et al., 1999).

Although corticospinal contributions to the observed RT reductions cannot be excluded completely (Alibiglou & MacKinnon, 2012; Marinovic & Tresilian, 2016), the significant differences in RT between the VSRT and VART/VRT conditions strongly suggest a dominant role of the RST. This aligns with findings from the study by Tapia et al. (2022), who demonstrated that loud auditory stimuli suppress corticospinal activity but enhance reticulospinal activity in the early post‐cue period, reinforcing the notion that the RST provides the majority of the descending drive during StartReact responses. Their computational modelling further supports the conclusion that a reticulospinal contribution of ≥60% is required to replicate the observed StartReact RT reductions.

To isolate the contribution of the RST further, we calculated the StartReact effect, focusing on the RT difference between the VART and the VSRT conditions to exclude general facilitatory effects of auditory stimulation. This subtraction‐based method provides a direct and interpretable measure of startle‐evoked enhancement in motor output. The StartReact effect was significantly different from zero across all three muscles, supporting active recruitment by the reticulospinal system. Contrary to prior studies suggesting greater RST activation for proximal muscles (Maslovat et al., 2023), the gain (Figure 2c) was significantly higher for the intrinsic hand muscle (1DI) compared with the more proximal muscles (FDS and biceps). This could be attributable to a targeted increase in the RST drive towards the hand, optimizing its contribution to executing a power grip. Direct intracellular recordings from motoneurons show that inputs from the RST are seen more frequently in intrinsic hand muscles than forearm flexors (Riddle et al., 2009). The convergence of respiratory and reticulospinal modulation at these transition points might reflect an evolved mechanism for optimizing motor performance during physiologically demanding states. Interestingly, Baker and Perez (2017) found in spinal cord injury patients that RST input depended on the task with higher input during power grips than during precision grips.

The respiratory phase significantly influenced RTs in the VSRT condition, where RTs were shortest during the transition from inspiration to expiration (I3–E1) for the 1DI and FDS muscles. This inspiration–expiration transition phase (Hülsmann et al., 2021; von Euler & Trippenbach, 1975), and the reverse (expiration–inspiration), represents critical moments in the physiological rhythm of the body, with notable effects on cortical and motor excitability (Nakamura et al., 2018; Zelano et al., 2016). Likewise, these respiratory transitions seem to modulate RST. As shown in Figure 4, it is evident that RST gain increases during transitions between inspiration and expiration, in addition to near the transitions from expiration to inspiration, and significant decreases in RST gain are observed mid‐expiration across all three muscles. This pattern highlights the dynamic modulation of the RST motor pathway excitability by the respiratory phase.

By pairing VNS with task‐specific rehabilitation, studies have shown significant improvements in motor function, suggesting that VNS triggers long‐term plasticity in motor circuits (Dawson et al., 2021; Engineer et al., 2019; Hays et al., 2016). Although cortical reorganization is frequently proposed as a primary mechanism of VNS‐induced recovery (Khodaparast et al., 2014), a more anatomically proximal motor circuit might also play a role. Specifically, the reticular formation and its motor output, the RST, might be modulated by VNS. Vagus nerve afferents project to the nucleus tractus solitarii in the brainstem, which connects to the reticular formation (Peyron et al., 1996; Ruggiero et al., 2000). By activating the reticular formation, VNS could influence RST activity, potentially driving motor recovery. Given the role of the vagus nerve in regulating breathing rhythms and the transition between inspiration and expiration (von Euler, 1983; von Euler & Trippenbach, 1975), VNS could potentially harness respiratory‐driven modulation to enhance RST activity and strengthen its contribution to motor recovery. Although direct effects of VNS on the RST remain to be demonstrated, this pathway represents a promising target for leveraging respiratory‐driven neural plasticity to optimize motor recovery.

The recent study by Choudhury et al. (2020) presents an intriguing application of RST modulation in motor rehabilitation. Their wearable device uses auditory–motor pairing to induce plasticity in the reticulospinal system, improving upper‐limb function in chronic stroke patients. Our findings complement this approach by highlighting the importance of respiratory‐phase transitions in modulating RST excitability. By incorporating respiratory‐phase timing into such interventions, specifically targeting stimulation to coincide with the transition from inspiration to expiration, we could, potentially, optimize the timing of therapeutic interventions, potentially enhancing motor recovery outcomes.

The enhanced reticulospinal drive during respiratory transitions, particularly during startle‐evoked responses, points to a novel mechanism by which fundamental physiological rhythms shape motor system function. In summary, this study provides evidence that respiratory rhythms significantly influence RST excitability, with transitions between respiratory phases serving as critical moments for modulating motor pathway activity. By combining these insights with existing evidence of RST plasticity and its role in motor recovery, our findings provide a foundation for future research aimed at integrating respiratory‐phase‐dependent modulation into neurorehabilitation strategies. Targeted interventions, such as respiratory‐phase‐aligned stimulation using wearable devices leveraging auditory–motor pairing, might harness these mechanisms to enhance motor recovery following corticospinal lesions. These advances could pave the way for personalized therapies that align with the natural rhythms of the body to maximize functional outcomes.

## AUTHOR CONTRIBUTIONS

Ruqayya Thawer, Stuart N. Baker and Boubker Zaaimi conceived and designed the study. Ruqayya Thawer and Boubker Zaaimi collected the data. Ruqayya Thawer, Stuart N. Baker and Boubker Zaaimi analysed the data. All authors contributed to data interpretation and manuscript preparation. All authors approved the final version of the manuscript and agree to be accountable for all aspects of the work in ensuring that questions related to the accuracy or integrity of any part of the work are appropriately investigated and resolved. All persons designated as authors qualify for authorship, and all those who qualify for authorship are listed.

## CONFLICT OF INTEREST

None declared.

## FUNDING INFORMATION

This work was supported by Aston University and Newcastle University. No external funding was received for this study.

## Data Availability

All data supporting the results presented in this manuscript are included within the figures. Additional data and analysis scripts are available from the corresponding author upon reasonable request
